# Supplementary material for: Cell Therapy Transplant Canada (CTTC) Consensus-Based Guideline 2024 for Management and Treatment of Chronic Graft-Versus-Host Disease and Future Directions for Development
Source: Curr Oncol. 2024 Mar 8;31(3):1426–44. doi: 10.3390/curroncol31030108 (PMC10968999; doi:10.3390/curroncol31030108)
Supplement: Supplementary file 1 [file curroncol-31-00108-s001.zip › curroncol-2848405-supplementary.pdf]

Supplementary Material

Table S1. Summary of Chronic GvHD systematic review, reprinted with permission from Ref. [11], 2024 American Society of Hematology.

No.	System/Others	Inquire/description
1	Skin	Skin feels tight or hard, increased dryness, pruritus, or looks different (i.e. new rash, papules, discoloration, shining scar-like, scaly)?
2	Sweat glands	Inability to sweat or to keep body warm?
3	Skin appendages	Loss of hair (scalp or body including brows or lashes), or nail changes (ridges or brittle, loss)?
4	Fasciae/Joints	Stiffness or pain in the wrists, fingers, or other joints?
5	Eyes	Eye dryness, sensitivity to wind or dry environments (air conditioning), pain?
6	Mouth	Oral dryness, taste alterations, sensitivities (spicy/carbonated drinks, toothpaste), ulcers/sores, pain?
7	Esophagus	Foods or pills gets stuck upon swallowing?
8	Lungs	Cough, dyspnea (on exertion or rest) or wheezing?
9	Genital tract	Vaginal dryness, pain, dyspareunia (female); pain or dysuria due to stenosis of urethra (male)?
10	Weight loss	Unexplained weight loss or inability to gain weight (pancreatic insufficiency or hypercatabolism)?

Adopted from *Blood* **2015**, 125, 606–615 [11].
